# Cyclic Nucleotide-Gated Channels Contribute to Thromboxane A_2_-Induced Contraction of Rat Small Mesenteric Arteries

**DOI:** 10.1371/journal.pone.0011098

**Published:** 2010-06-14

**Authors:** Yuk Ki Leung, Juan Du, Yu Huang, Xiaoqiang Yao

**Affiliations:** 1 Li Ka Shing Institute of Health Sciences, Chinese University of Hong Kong, Hong Kong, China; 2 School of Biomedical Sciences, Chinese University of Hong Kong, Hong Kong, China; 3 Department of Physiology, Anhui Medical University, He Fei, China; University of Cincinnati, United States of America

## Abstract

**Background:**

Thromboxane A_2_ (TxA_2_)-induced smooth muscle contraction has been implicated in cardiovascular, renal and respiratory diseases. This contraction can be partly attributed to TxA_2_-induced Ca^2+^ influx, which resulted in vascular contraction via Ca^2+^-calmodulin-MLCK pathway. This study aims to identify the channels that mediate TxA_2_-induced Ca^2+^ influx in vascular smooth muscle cells.

**Methodology/Principal Findings:**

Application of U-46619, a thromboxane A_2_ mimic, resulted in a constriction in endothelium-denuded small mesenteric artery segments. The constriction relies on the presence of extracellular Ca^2+^, because removal of extracellular Ca^2+^ abolished the constriction. This constriction was partially inhibited by an L-type Ca^2+^ channel inhibitor nifedipine (0.5–1 µM). The remaining component was inhibited by L-*cis*-diltiazem, a selective inhibitor for CNG channels, in a dose-dependent manner. Another CNG channel blocker LY83583 [6-(phenylamino)-5,8-quinolinedione] had similar effect. In the primary cultured smooth muscle cells derived from rat aorta, application of U46619 (100 nM) induced a rise in cytosolic Ca^2+^ ([Ca^2+^]_i_), which was inhibited by L-*cis*-diltiazem. Immunoblot experiments confirmed the presence of CNGA2 protein in vascular smooth muscle cells.

**Conclusions/Significance:**

These data suggest a functional role of CNG channels in U-46619-induced Ca^2+^ influx and contraction of smooth muscle cells.

## Introduction

Thromboxane A_2_ (TxA_2_) is an unstable prostanoid produced predominantly in platelets from prostaglandin H2 by thromboxane-A synthase. Functionally, TxA_2_ acts on the TP receptor (TxA_2_ receptor) to promote platelet aggregation [1] and to induce smooth-muscle contraction [2], [3]. Two main mechanisms underlie the TxA_2_-induced contraction of vascular smooth muscle. The first mechanism is the Ca^2+^ sensitization of contraction, which refers to a sensitized contractile response to a small rise in [Ca^2+^]_i_ [4]. This mechanism has been extensively studied in recent years. It is clear that the Ca^2+^ sensitization can be attributed to a reduced activity of myosin light-chain phosphatase, followed by a greater degree of phosphorylation of myosin light chain 20, leading to the sensitized contractile response to Ca^2+^ [5]. The second mechanism is related to TxA_2_-elicited increase in [Ca^2+^]_i_, which enhances the smooth muscle cell contraction via Ca^2+^-calmodulin-myosin light-chain kinase pathway. Relatively little is known about the detailed mechanism of how TxA_2_ elicits a [Ca^2+^]_i_ rise. Tosun et al. found that both L-type and non-L-type Ca^2+^ influx channels could account for thromboxane A_2_ receptor-mediated contraction in rat aorta, but the identity of the non-L-type calcium channel is unclear (1998). Evidence also shows that the Ca^2+^ sensitization process also requires Ca^2+^ influx, because removal of extracellular Ca^2+^ abolished the Ca^2+^ sensitization [2], [6].

Cyclic nucleotide-gated (CNG) channels are Ca^2+^-permeable nonselective cation channels. Six CNG isoforms have been identified, these includes four A subunits and two B subunits. CNGA1–A3 subunits may form functional channels on their own, while B and A4 subunits serve modulatory functions. In native cells, CNG channels usually form heterotetrameric complexes consisting of A and B subunits [7]. CNG channels are widely expressed in vascular tissues across species and vascular beds [8], [9]. Specifically, CNGA1 was found to be abundantly expressed in the endothelium layer, and also expressed in vascular smooth muscle layers but at a much lower level in guinea pig arteries [9]. In contrast, strong expression of CNGA2 channel was detected in both the endothelium and smooth muscle layers of human arteries [8]. Functionally, endothelial cell CNG channels play an important role in endothelium-dependent vascular dilation to a number of cAMP-elevating agents including adenosine, adrenaline and ATP [10], [11], [12]. However, up to the present, there is still no report on the functional role of CNG channels in smooth muscle cells.

In the present study, we tested the hypothesis that CNG channels may contribute to TxA_2_-induced Ca^2+^ influx and contraction in vascular smooth muscle cells. A stable TxA_2_ analogue U-46619 was used to induce contraction in the endothelium-denuded small mesenteric artery segments. This constriction was inhibited by L-*cis*-diltiazem and LY83583, two selective inhibitors for CNG channels, in dose-dependent manner. Immunoblot experiments found the expression of CNGA2 proteins in the primary cultured vascular smooth muscle cells. These data suggest a functional role of CNG channels in U-46619-induced Ca^2+^ influx smooth muscle contraction.

## Results

**Figure 1:**
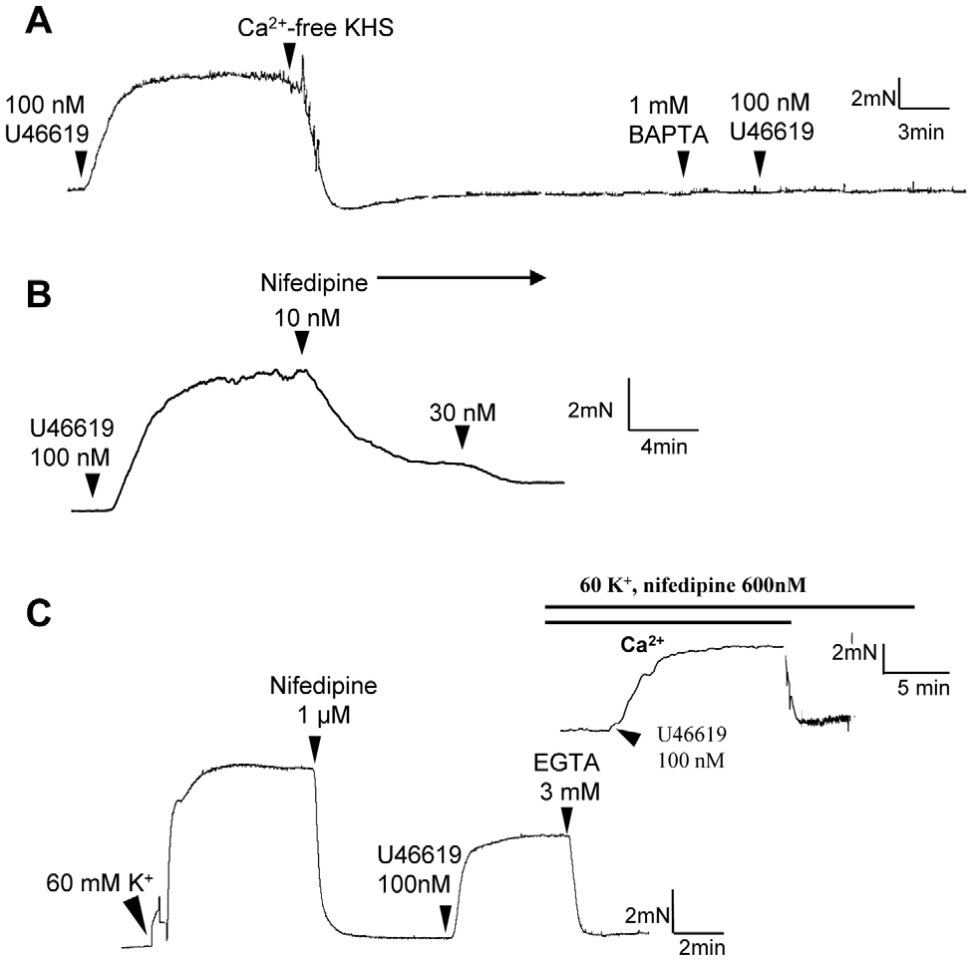
Representative traces of the tension developed in endothelium-denuded small mesenteric arteries. **A**. Addition of 1 mM BAPTA in Ca^2+^-free KHS prevented the U46619-induced contraction. **B**. Nifedipine significantly inhibited the U46619-induced contraction. **C**. 1 µM nifedipine was added to block the contraction induced by high K^+^ solution. After stabilization, U46619 was added to recontract the vessel, followed by the addition of 3 mM EGTA. Inset of C. similar to C, except that the U46619-induced contraction was followed by replacing the bath solution by a Ca^2+^-free KHS. n = 5.

Because TxA_2_ is an unstable compound, we used a stable TxA_2_ analogue U-46619 (100 nM) to induce contraction in isolated rat small mesenteric artery segments (Figure 1A). To limit the study to vascular smooth muscle cells, the endothelial layer was rubbed off with wire before all contraction studies. U-46619-induced vascular contraction in these artery segments depended upon Ca^2+^ influx, because U-46619 failed to induce contraction after chelation of extracellular Ca^2+^ with 1 mM BAPTA (Figure 1A). We next explored the identity of the Ca^2+^-permeable channels. In the vessels that were pre-contracted with U-46619 (100 nM), an L-type Ca^2+^ channel inhibitor nifedipine relaxed the vessels (Figure 1B), suggesting an involvement of L-type Ca^2+^ channels in U-46619-induced smooth muscle contraction.

**Figure 2:**
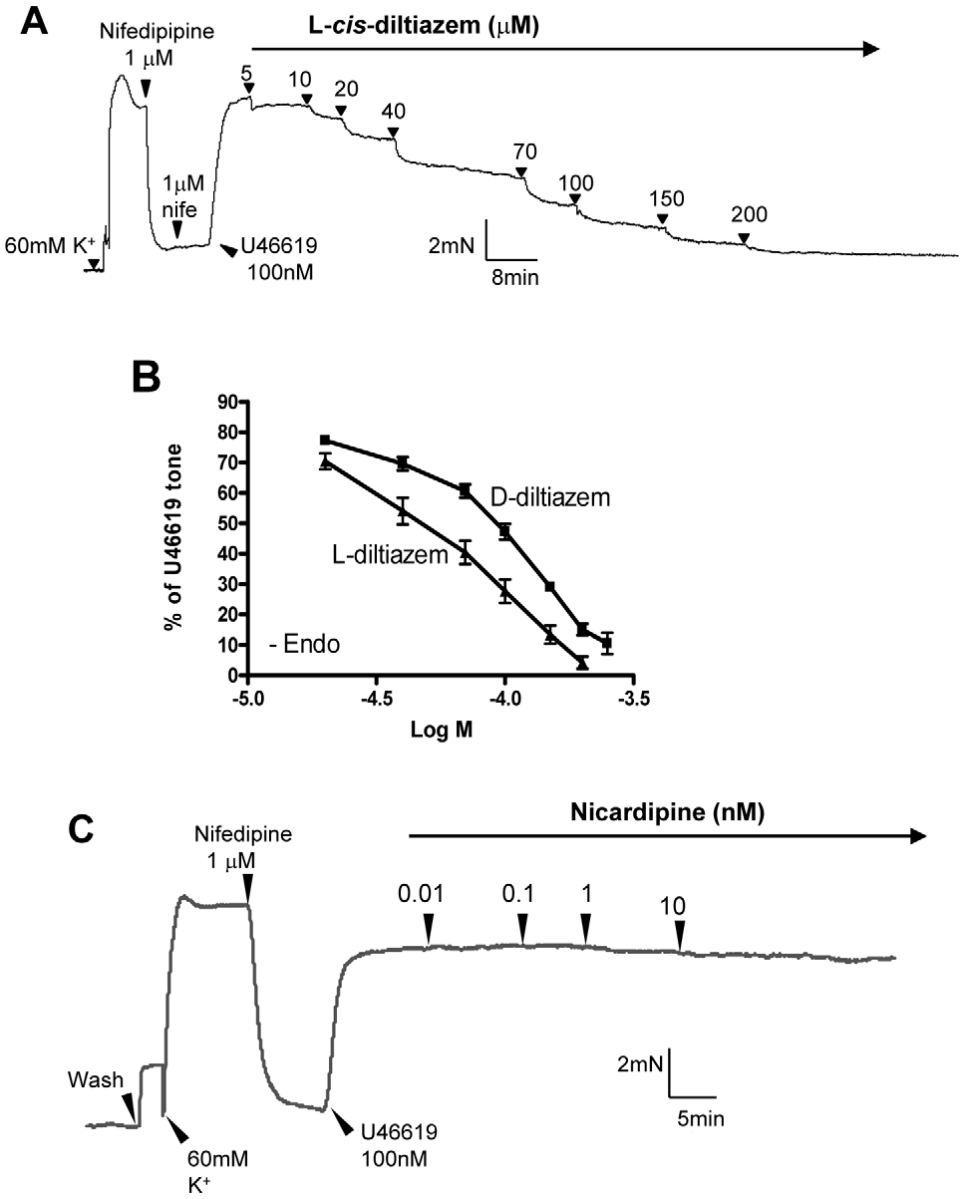
Effect of L-*cis*-diltiazem on the contraction induced by U46619. **A**. A representative trace of the tension developed in an endothelium-denuded small mesenteric artery. 1 µM Nifedipine was added to inhibit L-type Ca^2+^ channels. U46619 (100 nM) was added to recontract the vessel, which is followed by cumulative doses of L-*cis*-diltiazem. N = 5. **B**. A dose response curve showing the concentration dependent effect of L- and D-*cis*-diltiazem on contractions induced by U46619 after the blockage of L-type voltage-gated Ca^2+^ channels. Mean ± SE (n = 5). **C**. A representative trace of the tension developed in the ring showing that nicardipine had no additional effect on U-46619-induced contractions after the blockage of L-type voltage-gated Ca^2+^ channels by nifedipine. N = 4.

**Figure 3:**
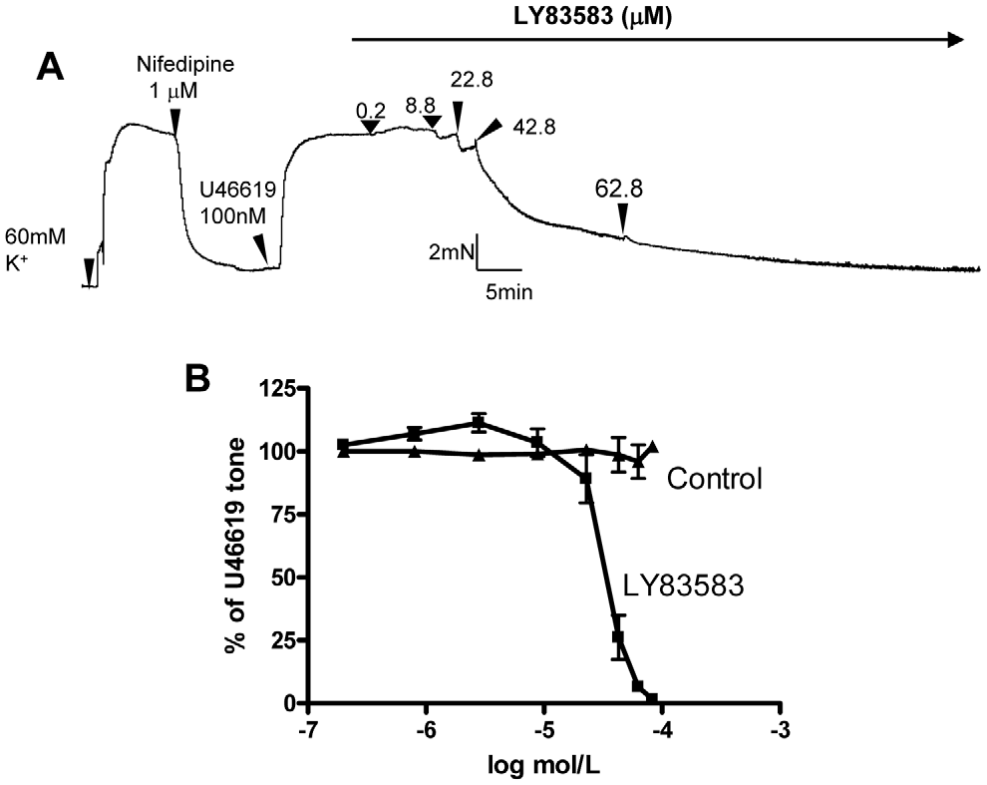
Effect of LY83583 on U-46619-induced vasoconstriction in an endothelium-denuded small mesenteric artery segment. **A**. A representative trace of the tension developed in the ring upon various treatments. **B**. A dose response curve showing the concentration dependent effect of LY83583 on contractions induced by U-46619 in the presence of L-type voltage-gated Ca^2+^ channels blocker. Mean ± SE (n = 4).

We next explored other Ca^2+^-permeable channels involved. In order to better resolve the component that was independent of L-type Ca^2+^ channels, arterial segments were first treated with nifedipine (1 µM) to inhibit L-type Ca^2+^ channels (Figure 1C). In our preparation, 1 µM nifedipine was sufficient for complete inhibition of L-type Ca^2+^ channels, because it could almost fully reverse the high K^+^ (60 mM)-induced contraction (Figure 2A). High concentration of K^+^ in extracellular bath causes membrane depolarization, which open voltage-gated L-type Ca^2+^ channels, resulting in vascular contraction [13]. After the inhibition of L-type Ca^2+^ channels with nifedipine, subsequent addition of U-46619 could still induce contraction (Figure 1C, Figure 2A). Again, the U-46619-induced contraction required Ca^2+^ influx, because it was reversed by removal of extracellular Ca^2+^ (Figure 1C inset) or addition of EGTA in the bath (Figure 1C). We next explored the possible involvement of CNG channels. As shown in Figure 2A, cumulative addition of L-*cis*-diltiazem (5–200 µM) caused a concentration-dependent inhibition of U-46619-induced contraction. D-*cis*-diltiazem had similar inhibitory effect on the contraction, though its effect was less potent than that of L-*cis*-diltiazem (Figure 2B). L-*cis-*Diltiazem is a highly selective inhibitor of CNG channels. It blocks all three functional types of CNG channels, including rod-type CNGA1, olfactory-type CNGA2 and cone-type CNGA3, at micromolar concentration [14], [15]. The drug exerts its effect from the cytoplasmic face of the channel; extracellular application is less effective [16], [17]. D-*cis*-diltiazem is the enantiomer of L-*cis*-diltiazem. D-*cis*-diltiazem is a well-known blocker of L-type Ca^2+^ channels. Both L-*cis*-diltiazem and D-*cis*-diltiazem are membrane-permeable [14], [15]. In our experiments, L-type Ca^2+^ channels were first blocked by sufficient concentration of nifedipine, thus the effect of L-*cis*-diltiazem and D-*cis*-diltiazem on the U-46619-induced contraction could not be attributed to L-type Ca^2+^ channels. As a precaution, we also tested the effect of nicardipine, which is another potent blocker of L-type Ca^2+^ channel (Figure 2C). Previously, it was shown that nicardipine at 1 nM could completely inhibit L-type Ca^2+^ channels [18]. In the present study, nicardipine up to 10 nM had no effect on the U-46619-induced contraction. These results confirm that the observed effect of L-*cis*-diltiazem was due to its action on CNG channels but not on L-type Ca^2+^ channels. We also tested another CNG channel blocker LY-83583 [19]. This agent also caused dose-dependent inhibition on U-46619-induced contraction (Figure 3).

**Figure 4:**
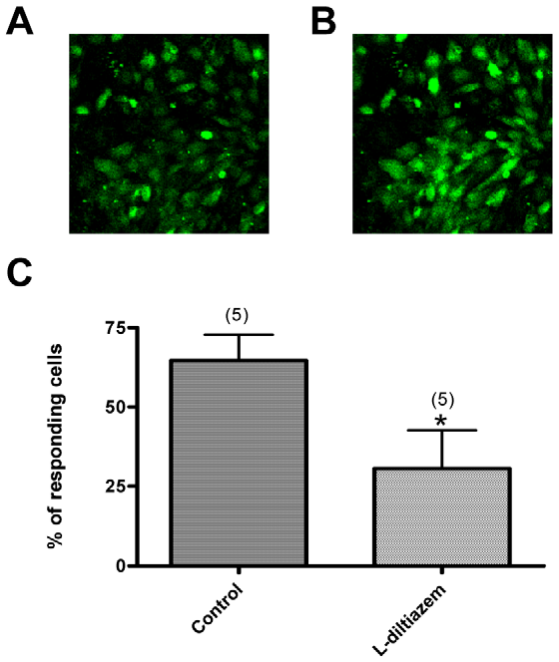
Effect of U46619 and L-*cis*-diltiazem on [Ca^2+^]_i_ in the primary cultured aortic smooth muscle cells. Cells were pretreated with 300 nM nifedipine to block L-type Ca^2+^ channels. **A**, **B**. Representative fluorescence images of cultured smooth muscle cells before (**A**) and after (**B**) 100 nM U46619. **C**. Summary of data showing the inhibitory effect of L-*cis*-diltiazem (100 nM) on U46619-induced [Ca^2+^]_i_ rises. Shown were the percentage of cells displaying U46619-induced [Ca^2+^]_i_ rises before and after U46619 challenge, Mean ± SE (n = 5). * p<0.05 as compared with the control.

U-46619-induced Ca^2+^ influx was studied in the primary cultured rat vascular smooth muscle cells. In these studies, the cells were incubated with nifedipine (300 nM) to inhibit L-type Ca^2+^ channels. This would allow us to better resolve the Ca^2+^ influx pathway that is independent of L-type Ca^2+^ channels. As shown in Figure 4A,B, U-46619 still induced a cytosolic Ca^2+^ rise in these cells. L-*cis*-diltiazem (100 µM) inhibited this Ca^2+^ rise, supporting a role of CNG channels in this Ca^2+^ rise.

**Figure 5:**
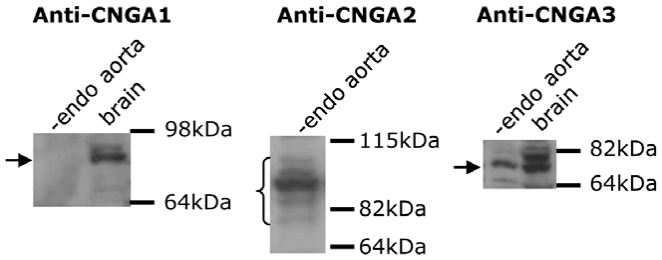
Western blotting showing the expression of CNGA2 and CNGA3 proteins in rat aortic smooth muscle cells. Shown were representative images from 3 independent experiments.

Immunoblots were used to examine the CNG channel isoforms that are expressed in vascular smooth muscle cells. Figure 5 shows that an anti-CNGA2 and an anti-CNGA3 antibody recognized respective proteins with expected molecular sizes, while the anti-CNGA1 antibody failed to detect expression of CNGA1 in the protein lysates prepared from rat aortic smooth muscles. Note that multiple bands in the immunoblot of CNGA2 proteins might represent the same proteins that were glycosylated at different degree [20].

## Discussion

TxA_2_ is implicated in many cardiovascular, renal and respiratory diseases [21]. Much of these problems is associated with TxA_2_-induced smooth muscle contraction, which leads to vascular constriction, ischemia, pulmonary hypertension and broncho-constriction [22], [23]. In the present study, we explored the mechanism of TxA_2_-induced vascular smooth muscle cell contraction. Our results showed that, in the absence of extracellular Ca^2+^, U46619 failed to constrict the isolated small mesenteric artery segments. In addition, removal of extracellular Ca^2+^ relaxed the artery segments that were preconstricted with U-46619. Furthermore, inhibition of L-type Ca^2+^ channels by nifedipine reduced the contractile response to U-46619. These data agree with the previous reports by others [2], [3], and support the notion that Ca^2+^ influx is required for U46619-induced vascular constriction and that L-type Ca^2+^ channels play a role in this constriction.

Because the inhibition of L-type Ca^2+^ channels by nifedipine only caused a partial inhibition of U46619-induced constriction, we next explored the possible involvement of CNG channels in U46619-induced constriction. In these experiments, arterial segments were first treated with nifedipine to block L-type Ca^2+^ channels in order to better resolve the component that was independent of L-type Ca^2+^ channels. Under this condition, U-46619-induced contraction was found to be inhibited by L-*cis*-diltiazem in a dose-dependent manner. L-*cis*-diltiazem also inhibited U-46619-induced cytosolic Ca^2+^ rise in the primary cultured vascular smooth muscle cells. The inhibitory action of L-cis-diltiazem suggests the involvement of CNG channels in U-46619-induced Ca^2+^ rise in smooth muscle cells and subsequent vascular constriction. This hypothesis was supported by the LY83583 experiments, in which LY83583 also caused a dose-dependent inhibition of U-46619-induced vascular constriction. LY-83583 is a less selective agent and it inhibits both CNG channels and soluble guanylate cyclase at similar concentrations [19]. However, we reason that the inhibitory action of LY-83583 on U-46619-induced vascular constriction should be due to CNG channels but not to soluble guanylate cyclase, because an inhibition of soluble guanylate cyclase would result in contraction instead of the observed relaxation (Figure 5).

CNG channels are widely expressed in different vascular tissues including cerebral and coronary arteries [8], [9], [24], [25]. There are three functional subunits CNGA1-A3. All these three subunits were reported to be expressed both in vascular endothelial cells and vascular smooth muscle cells [8], [9], [24]–[26]. Previous study also suggested that CNGA1 expression in vascular smooth muscle is very low, because RT-PCR could detect CNGA1 in cultured vascular smooth muscle cells but Western blot and in situ hybridization failed to detect CNGA1 in vascular tissues [9], [24]. In the present study, we found the expression of CNGA2 and A3, but not A1, in the protein lysates from rat vascular smooth muscle cell layers. These data are consistent with the previous reports, and suggest that CNGA2 or A3, but not CNGA1, are more likely to be involved in the U-46619-induced vascular constriction. Note that unlike other CNG isoforms [16] which are insensitive to D-*cis*-diltiazem even at mM concentration, olfactory-type CNGA2 is inhibited by D-*cis*-diltiazem though with much less potency compared to that of L-*cis*-diltiazem. In our experiments, high concentration of D-*cis*-diltiazem inhibited U-46619-induced vascular constriction with the dose-response curve mirrors that of olfactory-type CNGA2 reported by Frings et. al. [15]. These data suggest olfactory-type CNGA2 to be a more likely candidate that is involved in the U-46619-induced vascular constriction.

In conclusion, TxA_2_-induced vascular contraction was inhibited by two CNG channel inhibitors L-*cis*-dialtiazem and LY-83583. Our data suggest that CNG channels, olfactory-type CNGA2 in particular, contribute to TxA_2_-induced Ca^2+^ influx in vascular smooth muscle cells.

## Materials and Methods

### Ethics statement

The animal study was conducted in conformity with the *Guide for Animal Care and Use of Laboratory Animals* published by the US National Institute of Health. The experiments were approved by the Department of Health of Hong Kong and carried out by investigators licensed (06-3indh/orhi/821pt.5) under section 7 of the *Animals (Control of Experiments) Ordinance (Cap. 340)*.

### Blood vessel preparation

Mesenteries with supplying blood vessels removed from adult Sprague Dawley rats (250 g–300 g) killed by carbon dioxide overdose were placed into Krebs-Henseleit solution (KHS) (in mM): 118 NaCl, 4.7 KCl, 2.5 CaCl_2_, 1.2 MgSO_4_, 25.2 NaHCO_3_, 1.2 KH_2_PO_4_, and 11.1 D-glucose. Second order small mesenteric artery ring segments of about 3 mm in length were isolated and cleared of adhering fatty tissues. Each segment was mounted in the tissue chamber of a Multi Myograph System (Danish Myo Technology, Aarbus, Denmark) with two wires passing through the lumen. The tissue chambers contained KHS that was constantly bubbled with 95% O_2_ plus 5% CO_2_ and maintained at 37°C throughout the duration of the experiment. Endothelium was removed mechanically by gently rubbing the luminal surface with a piece of stainless steel wire. Each ring was stretched to an initial tension of 1 mN and the changes in tension were recorded in a myograph. The rings were precontracted twice in 60 mM K^+^ KHS and then contracted with 2 µM phenylephrine, followed by 1 µM acetylcholine to ascertain the completeness of endothelium removal. Only those with less then 5% relaxation were considered as endothelium denuded. The rings were then washed three times in normal KHS.

### Measurement of isometric force

To study if Ca^2+^ influx is required for U46619-induced contraction, 100 nM U-46619 was added to initiate contraction in the rings. After that, the rings were washed three times with Ca^2+^-free KHS and incubated in 1 mM BAPTA before the rings were recontracted with the same concentration of U-46619. In some experiments, EGTA (3 mM) was added or extracellular Ca^2+^ was removed after the U-46619-induced tension became stable (n = 4).

To assess the effect of L-*cis*-diltiazem on U-46619-induced contraction, the bathing solution was changed to 60 mM K^+^ solution to open the voltage-gated Ca^2+^ channels, followed by an addition of 1 µM nifedipine to block L-type voltage-gated Ca^2+^ channels. After the tone returned to basal level, which is an indication that most, if not all, of the voltage-gated channels were blocked, the vessels were recontracted with 100 nM U-46619. Cumulative doses (from 20 µM to 200 µM) of L-*cis*-diltiazem, a specific CNG channel blocker, were added to assess the role of CNG channels in the contraction. The concentration-dependent relaxant effect of D-*cis*-diltiazem was also examined for comparison. A control experiment running in parallel without the addition of diltiazem showed that the U-46619-induced contraction was sustained during the experimental time period.

### Cell culture

The primary cultured rat aortic smooth muscle cells were isolated from SD rat aorta as described elsewhere [27]. Briefly, the adherent adventitia layer was peeled off before the aorta was cut into small pieces and digested with 0.1% collagenase in Ca^2+^-free PSS (in mM: 55 NaCl, 80 sodium glutamate, 5.6 KCl, 10 HEPES, 2 MgCl_2_, 10 glucose, pH 7.4) for 25 min at 37°C under vigorous shaking. The tissues were then rinsed several times in PSS to remove the collagenase. Smooth muscle cells were dissociated from the tissues by pipetting up and down using a Pasteur pipet. The cells were seeded on coverslip and grown in 80% DMEM and 20% FBS with 1% antibiotic-antimicotic for a week before Ca^2+^-imaging experiments.

### [Ca^2+^]_i_ measurement

Cultured cells were loaded with 10 µM Fluo-4/AM for 20 min in dark at 37°C in culture medium. Then the cells were briefly washed and bathed in a normal physiological saline solution (NPSS) that contained in mM: 140 NaCl, 5 KCl, 1CaCl_2_, 10 glucose, 5 Hepes, pH 7.4. The experimental chambers were placed on the stage of an inverted microscope (Olympus IX81). Fluorescence was measured using the FV1000 laser scanning confocal imaging system. The excitation wavelength was at 488 nm and the fluorescence signals were collected using a 515 nm long pass emission filter. Data analysis was performed with FV1000 software. Changes in [Ca^2+^]_i_ were displayed as a ratio of fluorescence relative to the fluorescence before the application of U-46619.

### Western blotting analysis

Thoracic aorta was isolated from Sprague Dawley rats (250–300 g) and placed in PBS immediately. The surrounding connective tissue was carefully trimmed off and endothelial cells removed by rubbing with a piece of cotton. The aorta was then homogenized in lysis buffer containing 50 mM Tris–base, 150 mM NaCl, 50 mM NaF, 1% Nonidet P-40, 0.5% sodium deoxycholate, pH 7.5, with addition of the protease inhibitor cocktail tablets (Roche) and centrifuged at 10,000 *g* for 5 min at 4°C. The supernatant was collected, and the protein concentration was quantified using Bradford reagent (BioRad, Hercules, CA). Samples (50 g protein/lane) were subjected to SDS-polyacrylamide gel electrophoresis, and transferred to polyvinylidene difluoride membrane. Antibodies against CNGA1 (1∶200, Santa Cruz) or CNGA2 (1∶1000, polyclonal, directed against residues 559–664 of CNGA2) or CNGA3 (1∶200, Alamone lab) were incubated with the membrane overnight at 4°C. The anti-CNGA2 polyclonal antibody was self-raised following a previous published protocol [20]. Immunolabeled membranes were subjected to probing with horseradish peroxidase-linked secondary antibodies, and visualized by chemiluminescence using ECL kit (GE Healthcare, Little Chalfont, Buckinghamshire, UK).

### Materials

Chemicals. Acetylcholine, nifedipine, ethylene glycol tetraacetic acid (EGTA), 1,2-bis(o-aminophenoxy) ethane-N,N,N',N'-tetraacetic acid (BAPTA) and D-*cis*-diltiazem were purchased from Sigma Aldrich, L-*cis*-diltiazem from Biomol International and U-46619 from Calbiochem. U-46619, BAPTA and nifedipine were dissolved in DMSO and others in distilled water.
